# Barriers and Facilitators to Implementing a School-Based Social and Emotional Learning Program for Rural Children in China: A Qualitative Study Using the Consolidated Framework for Implementation Research

**DOI:** 10.1007/s10488-025-01460-z

**Published:** 2025-07-15

**Authors:** Linyun Fu, Yibin Yang, Alida Bouris

**Affiliations:** 1 Crown Family School of Social Work, Policy, and Practice, The University of Chicago, Chicago, USA; 2 School of Social Work, Boston University, Boston, USA

**Keywords:** Social and Emotional Learning, Implementation barriers and facilitators, School mental health, Teacher, Rural children

## Abstract

Rural children in mainland China, particularly those whose parents migrate for economic opportunities, face distinct mental, emotional, and behavioral (MEB) challenges. With few mental health professionals available to address rural student’s MEB needs, task-shifting approaches that rely on teacher-led interventions, such as the evidence-based Social and Emotional Learning (SEL) program, are a promising alternative. However, little is known about the implementation determinants of teacher-led SEL programs in rural China, a key gap for sustaining and scaling effective programs. The present study qualitatively explored the contextual determinants of implementing the Positive Growth Curriculum (PGC), an efficacious, teacher-led SEL program delivered to 648 fifth graders in two rural schools in southwest China. We conducted in-depth interviews with all *N* = 15 schoolteachers and administrators who implemented PGC, with questions guided by the Consolidated Framework for Implementation Research. Interviews were audio recorded and two independent coders conducted Rapid Qualitative Analysis. Codes were organized into four major themes detailing how contextual determinants shaped the acceptability, appropriateness, feasibility, fidelity, and sustainability of PGC. Findings indicated that innovation facilitators, e.g., relative advantage, low complexity, intervention design, and perceived effectiveness, facilitated PGC acceptability and appropriateness (Theme 1) and implementation strategies, e.g., training, facilitation and champions, were vital for ensuring fidelity (Theme 2). However, despite high teacher motivation and buy-in, feasibility and sustainability were threatened by inner setting pressures and administrative burdens that prioritize student’s academic success over their SEL (Theme 3) and a lack of community buy-in and low governmental funding for SEL initiatives, two outer setting determinants (Theme 4). These barriers led some teachers to question if they are the optimal deliverers for SEL curricula. Potential PGC adaptations are discussed, as are possible implementation strategies and adjunctive interventions to sustain and scale effective teacher-led programs that can support rural students’ MEB health and academic success.

**Supplementary Information:**

The online version contains supplementary material available at 10.1007/s10488-025-01460-z.

## Introduction

In the past few decades, Mainland China has had an alarmingly high number of children experiencing mental, emotional, and behavioral (MEB) challenges. Despite limited national research on children’s mental health, a recent nationwide epidemiological study involving 73,992 school-aged children aged 6–16 indicated that the overall prevalence of common mental health disorders, such as attention-deficit and disruptive disorders, anxiety disorders, and depressive disorders, was approximately 18% (Li et al., 2022). Existing evidence from regional studies also indicated that the prevalence of mental health disorders among children and youth in China ranged between 9.69 and 16.22% (Guan et al., 2010; Liu et al., 1999; Qu et al., 2015; Yang et al., 2014), and may have worsened after the COVID-19 pandemic (Liu et al., 2020; Zhang et al., 2020). For example, a recent systematic review identified the pooled prevalence of depression and anxiety among children and adolescents was 22% and 25%, respectively (Chai et al., 2021). With an estimated 298 million child population under the age of 18, accounting for nearly 21.1% of the country’s total population (National Bureau of Statistics of China, United Nations Children’s Fund China, & United Nations Population Fund China, 2023), these data indicate that a large number of children and youth may benefit from programs designed to address their mental, social, and academic well-being.

Among all children and youth, those living in rural areas, especially the children of work-away parents, experience a disproportionate burden of MEB challenges (Wang et al., 2019). In the present paper, we refer to rural children with one or both parents living and working away from the family home as children of ‘work-away’ parents instead of the commonly used ‘left-behind children’ in order to foreground the economic conditions that shape parents’ decisions to migrate in search of better work and economic opportunities and to reduce the stigma associated with characterizing children in this way (Fu & Zhu, 2020). A number of studies have found that rural children of work-away parents are more likely than their peers to develop MEB challenges, such as emotional distress, depressive symptoms, conduct problems, and low self-esteem (Guo et al., 2015; Hu et al., 2014; Liang et al., 2017; Tang et al., 2018; Zhang et al., 2019; Zhao & Yu, 2016). Consequently, if not receiving timely, evidence-based interventions, rural children of work-away parents may also be at a greater risk than their counterparts of experiencing poorer long term quality of life outcomes, including poorer physical, social, and academic functioning and performance (Jia et al., 2010; Huang et al., 2015). According to the latest data from the Ministry of Civil Affairs, there are currently over 69 million children of work-away parents living in rural areas of mainland China (UNICEF, 2020), mostly from the central or western regions as a large portion of the rural population migrates for work (Lu & Xia, 2016).

Western-oriented interventions that rely on talk therapies experience several barriers to implementation in rural China, including a nascent mental health infrastructure, a dearth of trained professionals, and a lack of general understanding regarding the importance of mental health (Wu & Pan, 2019). These barriers are common in many low and middle income countries, and global mental health experts recommended task-shifting, e.g., the practice of having non-professionals deliver mental health services, as a viable approach to addressing gaps in mental health services (Bolton, 2019; Philip & Chaturvedi, 2018; Purgato et al., 2020). For school-based children, task-shifting the delivery of mental health programming to teachers is a promising alternative (Fazel et al., 2014; Vanderburg et al., 2022b). In China, researchers have examined task-shifting of mental health support to teachers at the University level, finding that while faculty are motivated to support students, they face competing demands related to high teaching workloads (Hou et al., 2024). Faculty also described challenges with stigma, not feeling adequately prepared to address major mental health issues, and a lack of intersectoral coordination with formal mental health providers (Hou et al., 2024).

Despite these barriers, task-shifting remains a promising approach for supporting student mental health in China (Shi & Leuwerke, 2010), especially given recently passed legislation that prioritizes improving child and adolescent mental and physical health (Bao et al., 2019). As part of the Healthy China Action plan, the government released the Mental Wellbeing of Children and Youth Action Plan (2019–2022), which stresses the importance of preventive interventions to promote young people’s mental health across multi-system levels. Of particular relevance for supporting the task-shifting of mental health programming to teachers, the plan stipulates that schools must offer mental health services to students at all grade levels, with teachers playing an essential role in coordinating and offering timely mental health-focused services and supports (e.g., psychoeducation, counseling, support groups) to students in vulnerable situations, including those whose parents work away from home (National Health Commission of the People’s Republic of China, 2019).

One evidence-based program that offers considerable promise for task-shifting to teachers is built upon the Social Emotional Learning (SEL) framework, which seeks to cultivate five social-emotional competencies among children and adolescents: (a) self-awareness, (b) self-management, (c) social awareness, (d) relationship skills, and (e) responsible decision-making (Collaborative for Academic, Social, and Emotional Learning [CASEL], 2021). In addition, the SEL framework seeks to support young people by establishing systemic school-family-community partnerships (CASEL, 2021). Several meta-analyses have demonstrated the effectiveness of SEL-focused programs for improving social and emotional skills (Durlak et al., 2011, 2022) and academic performance (Corcoran et al., 2018), and for reducing internalizing and externalizing behaviors, such as depression, anxiety, stress, and aggression (Bierman et al., 2010; Murano et al., 2020; Cipriano et al., 2023).

The teacher-led model in SEL is well-suited for rural Chinese schools in several aspects. First, schools are a powerful developmental context for children, offering not only educational opportunities but also social and emotional support. This role is especially crucial for rural children of work-away parents (Fu & Zhu, 2020), for whom schools often become the most proximal and influential environment (Wen & Lin, 2012). In addition, the current policy context creates an opportunity for the potential adoption of SEL programs that can be led by teachers, making it more practical to implement these initiatives within existing school frameworks. Third, due to the scarcity of mental health resources and the limited funding that can be used to hire traditional mental health professionals such as child psychiatrists and pediatricians in rural school settings (Wu & Pan, 2019), the task of promoting students’ mental health is most often assumed by a current full-time teacher (Fu et al., 2024; Shi & Leuwerke, 2010), who are serving the role as non-traditional mental health providers. Therefore, training current school teachers to deliver SEL content may represent a more feasible and sustainable approach to addressing rural students’ mental health needs.

To date, few SEL programs have been developed for the Chinese context, with most research occurring in the United States (U.S.), United Kingdom, and Australia (Cipriano et al., 2023; Santos et al., 2023). However, as noted by Durlak et al. (2011), the quality of implementation processes is critical for understanding the effectiveness of SEL programs, in which both effectiveness and implementation outcomes could be negatively impacted if implementation problems are not properly monitored and resolved. Research has identified multi-level factors, i.e., implementation determinants, that can affect the successful implementation of teacher-led SEL programs in schools. For example, Eisman and colleagues (2022) qualitatively explored the factors that affected fidelity to *Michigan Model for Health (MMH)*, a CASEL-recognized evidence-based program (EBP) implemented by high school health teachers. In interviews with 23 teacher-implementers, Eisman et al. (2022) found that while teachers were motivated to support their students and appreciated the range of curricular activities, they also perceived that *MMH* content was misaligned with student’s social and emotional needs. The perceived lack of curricular fit was particularly acute for students with pre-existing MEBs, and may have contributed to teacher adaptations that lowered fidelity to the program (Eisman et al., 2022).

Other studies examining the implementation of evidence–based SEL programs have found that having a school culture that supports SEL is a key implementation facilitator (Domitrovich et al., 2019; Lee & Simmons, 2022), as is having supportive school administrators (Ransford et al., 2009). In addition, research underscores the importance of teachers’ own social and emotional competencies, as well as their self-efficacy to deliver SEL programs (Jennings & Greenberg, 2009; Lendrum et al., 2013; Ransford et al., 2009; Schonert-Reichl, 2017). Of particular importance given the increasing demands placed on teachers, stress and burnout remain major barriers to effective implementation (Jennings & Greenberg, 2009). In their evaluation of *Promoting Alternative Thinking Strategies* (PATHS), Ransford et al. (2009) found that teacher burnout was negatively correlated with self-efficacy, as well as teacher’s self-reports of program dosage and implementation quality. Similarly, Collie et al. (2015) found that while teachers from different schools demonstrated similarly high levels of commitment to implementing SEL programs, many reported feeling great challenges, resistance, and high job stress due to little or no systemic support at school. Other factors, such as relationships between implementers and stakeholders across different systems (i.e., school colleagues, students’ families, district leaders) and community-level resources and politics may also affect teachers’ experience of SEL program implementation within schools (Durlak & DuPre, 2008; Mischenko et al., 2022).

Given the relative dearth of research on SEL programs in China, much of the extant literature has focused on evaluating the efficacy of SEL programs in school settings (An et al., 2021; Fu et al., 2024; Wang et al., 2016), with less emphasis on the implementation determinants of these programs. However, available research indicates that similar contextual dynamics may affect the ability of teachers to adopt, implement and sustain SEL programs. For example, in a sample of kindergarten teachers from the southern province of Fujian, Zong et al. (2024) found that SEL knowledge, attitudes, and beliefs were higher among teachers with less than six years of teaching experience and more advanced education. Follow-up interviews revealed that while teachers had a foundational understanding of SEL content, most needed professional training and support to translate that knowledge into effective SEL programming and skill development with students (Zong et al., 2024). Due to the considerable disparities in education resources and quality between urban and rural areas in mainland China (Lei et al., 2018), many teachers in rural schools face significant challenges, such as large classes sizes and a heavy workload (Fu & Zhu, 2020; Tang, 2020), which may lead to increased burdens and barriers for implementing SEL programs. Indeed, in a separate study with primary and secondary teachers in Singapore, Ee and Cheng (2013) found that some teachers struggled with integrating SEL content into classroom lessons, as their primary focus was on academically preparing students. Hou et al. (2024) identified this same barrier among University faculty who participated in a task-shifting program to address college student’s mental health in China. However, both university faculty and elementary school teachers have noted the need for ongoing training and support to integrate SEL support into their routine work (Ee & Cheng, 2013), implementation strategies that have been successfully utilized to support SEL program implementation in other contexts (e.g., Eisman, Palinka et al., 2022; Ransford et al., 2009).

### Purpose of Present Study

While existing studies highlight the potential factors that can support teacher-led implementation of SEL programs in China, little research has examined the actual perspectives of teachers involved with delivering a SEL program. In addition, much of the current work has focused on teachers working in urban areas, with less attention to teachers in rural schools, including those tasked with supporting the academic and mental well-being of the children of work-away parents. The present study seeks to address these gaps by qualitatively exploring teacher and administrator perspectives on the implementation of the Positive Growth Curriculum (PGC), a SEL program that was tested in a quasi-experimental study with 648 fifth graders in two rural elementary schools in Chongqing (Fu et al., 2024), a province in Southwest China where 40–50% of children are estimated to have work-away parents (Beh & Ye, 2012; UNICEF, 2015). Through interviews with teachers and administrators who delivered the PGC, we sought to understand how contextual determinants, including the implementation strategies used to support teachers and school administrators to implement PGC, shaped key implementation outcomes of acceptability, appropriateness, feasibility, fidelity, and sustainability.

### The Positive Growth Curriculum Program

The PGC program was developed by the RICI (日慈 in Chinese) Foundation, a non-governmental organization based in mainland China focused on improving the mental well-being and academic achievements of children living in rural areas (RICI Foundation, 2022). A full discussion of the curriculum is outside the scope of the present paper but we briefly describe it here (Fu et al., 2024). PGC was specifically designed for rural elementary school students to enhance four key social-emotional skills: (a) self-awareness, (b) social awareness, (c) relationship skills, and (d) self-management. In addition to these outcomes, the program sought to reduce mental health symptoms, such as depression, anxiety, and antisocial behaviors, and to improve children’s academic performance. Because social-emotional competencies vary across cultural contexts (Hayashi et al., 2022), the curriculum was adapted to be culturally sensitive to rural Chinese children following guidelines from Bernal et al. (1995). The first and second authors of this paper were both members of RICI’s SEL curriculum development team.

With few school-based mental health professionals in China (Wu & Pan, 2019), the PGC was specifically designed to be delivered by teachers. The curriculum consists of eight 45–60 min sessions that teachers in each participating school deliver to fifth grade students on a weekly basis in the classroom setting. Curriculum modules were multi-modal, consisting of didactic lessons, animated clips, videos and art-based activities, with individual and group-based opportunities to practice SEL skills (Fu et al., 2024). Given teachers’ varied professional and personal responsibilities, there was no designated time for PGC delivery; instead, teachers could choose the most suitable time to deliver the program on a weekly basis. From March to June 2022, 13 teachers and 2 school administrators delivered the PGC to 648 students in two elementary schools. Outcome analyses indicated that PGC was efficacious for improving students’ overall self-reported social and emotional competencies, and demonstrated significant improvements in three SEL subdomains: (a) relationship skills, (b) self-awareness, and (c) social awareness (Fu et al., 2024). In addition, subgroup analyses indicated that intervention effects were more pronounced for the children of work-away parents, with children and teachers reporting high satisfaction with and acceptability of the program (Fu et al., 2024).

*Implementation strategies to support teacher delivery of PGC*. The RICI team utilized several implementation strategies designed to support teachers to implement PGC with fidelity. Table 1 lists the primary implementation strategies, operationalized according to Proctor et al. (2013) and harmonized to the Expert Recommendations for Implementation Change (ERIC) (Powell et al., 2015). The strategies included: (a) Identifying and preparing champions: Prior to implementation, the development team identified and contacted the dean of moral education (*deyu*) in each school and invited them to serve as a champion. In China, moral education is designed to cultivate and promote students’ development of political ideology, moral values, and citizenship (Li et al., 2004; Xue & Li, 2020). Teachers who serve as the dean of moral education are often tasked with overseeing the policies, curriculum, and programs concerning students’ moral development. Because the dean works closely with teachers and families within schools, they are ideally positioned to champion a new innovation. As PGC champions, deans were asked to select and recruit teachers interested in facilitating PGC and to disseminate information on PGC to teachers. Both deans pledged their commitment to serve as curriculum administrators in order to facilitate PGC implementation; (b) Distributing materials and conducting dynamic training: To improve teacher knowledge of SEL and increase their self-efficacy to deliver the curriculum, the PGC development team provided a full-day in-person training session to all implementing teachers at the two selected schools. The training included detailed lectures on SEL and students’ mental health, and provided teachers with curriculum manuals, toolkits, and other supporting materials. In addition, members of the development team also presented a 25-minute simulated teaching session, focusing on certain units of the PGC; (c) Facilitation: To support teacher’s self-efficacy and problem-solving skills and to support fidelity, RICI provided a multi-component facilitation strategy consisting of an online learning collaborative, technical assistance from RICI staff, and a fidelity check system. For the learning collaborative, teachers were invited to join a designated online chat group on WeChat (similar to WhatsApp in the U.S.). In addition to providing a space where teachers could collaboratively brainstorm effective teaching strategies, RICI members also joined the collaborative in order to provide ongoing technical assistance, offering pointers and answering questions in real-time. To assess fidelity, teachers submitted session-related documents (e.g., class notes, photos) after the completion of each module through an online system. The development team then reviewed the documents submitted by teachers and conducted regular individual check-ins with all participating schoolteachers to ensure that all curriculum sessions were being implemented as planned and that individual concerns regarding specific components or activities were addressed properly; (d) Involving executive boards: Members from the administrative team within the local schools, including school principals and curriculum administrators, were invited to actively participate in various stages of the PGC’s implementation. These essential staff members supervised the implementation process and offered vital administrative assistance to all participating school teachers (Fu et al., 2024); and (e) Providing incentives: With support from a nationwide charitable foundation, each participating teacher was offered a cash incentive of 100 RMB (equivalent to $13 U.S. dollars) upon the successful completion of each SEL session, with a total amount of 800 RMB ($110 U.S. dollars equivalent).

**Table 1:** Implementation strategies to support teachers in positive growth curriculum (PGC)

Strategy	Operational Definition	Actor	Dose
Identify and Prepare Champions	Identify and prepare the dean of moral education from each school to serve as the curriculum administrator to provide dedicated support during the implementation	Dean of Moral Education (*deyu*)	One time before baseline
Distribute Materials and Conduct Dynamic Training	Provide comprehensive in-person training for teachers, covering essential mental health knowledge and curriculum instructions	Curriculum Development Team	One full-day training at baseline
Facilitation	Provide multi-component facilitation strategies that include online learning collaborative, technical assistance from RICI staff, and a fidelity check system to ensure fidelity and resolve challenges during the curriculum implementation	Curriculum Development Team	Ongoing
Involve Executive Boards	Get staff from the school administrative team involved throughout the curriculum implementation and provide ongoing supervision and support to teachers	School Administrative Team	Ongoing
Provide Incentives	Set up a system of allocated funds as incentives and provide them to teachers upon the successful completion of each SEL unit	RICI Foundation	One time at post-completion

## Methods

### School and Participant Characteristics

The two elementary schools that implemented the PGC curriculum were rural town schools, each with an average student population of around 3,000. More than 75% of the students at these schools have at least one parent who works away in cities. Both schools are centrally located within rural town areas, surrounded by mountains in southwest China. All 13 fifth-grade classrooms across the two schools, totaling 648 students, participated in the SEL program.

Given the need to minimize teacher burden and provide teachers with adequate time to reflect on implementation, individual interviews were conducted during the summer, one month after the conclusion of PGC. The first author invited all program implementers, including 13 teachers and two administrators who attended the pre-implementation training and successfully implemented all PGC program sessions, to participate in an individual in-depth interview. All but one study participant identified as female, with a mean age of approximately 40.53 years (SD = 7.26) and a classroom size of around 50 students. Amongst all study participants, over half (*n* = 8) also served as the role of banzhuren (homeroom teacher) in the class. All study participants agreed to participate in the study and provided written consent. Table 2 presents the demographic characteristics of the participants in this study.

**Table 2:** Demographic characteristics (*N* = 15)

	Percent (*n*) or Mean (SD)
% Female	14 out of 15 (93.33%)
Mean age (SD)	40.53 (7.26)
Mean SEL class size (SD)	49.85 (4.88)
% Banzhuren of the class they taught SEL	8 out of 13 (61.53%)
% Teacher	13 out of 15 (86.67%)
% Administrators	2 out of 15 (13.33%)

### Data Collection

The first author interviewed participants using an interview protocol following the Consolidated Framework for Implementation Research (CFIR), a meta-theoretical framework derived from 19 implementation theories and models that identifies multi-level determinants that can shape the adoption of an evidence-based practice, program or policy (EBP) (Damschroder et al., 2009, 2022). First developed in 2009, the CFIR has become one of the most widely used determinant frameworks in implementation science, including research examining the implementation of school-based programs (McLoughlin et al., 2022; Richmond et al., 2020). Damschroder and colleagues (2022) updated the CFIR based on user feedback, with a focus on better situating recipient needs in the framework. The updated framework now has 48 constructs and 19 sub-constructs across five major domains: (a) the innovation characteristics of the EBP that is being implemented, e.g., the evidence-base, design content, complexity and relative advantage of the Positive Growth Curriculum, as well as it’s adaptability and cost, (b) the inner setting where the EBP is being carried out, e.g., features of the two elementary schools implementing PGC, including the culture, relational connections in the school, the tension for change, mission alignment between the school and the PGC, resources available to support PGC, and having access to knowledge about PGC; (c) the outer setting encompassing constructs exogenous to the setting where the EBP is being done, e.g., local values and beliefs about SEL, local and national policies regarding education and SEL, partnerships and connections, financing, external pressure and large scale critical incidents like the COVID-19 pandemic; (d) the characteristics of the individuals who deliver, support and receive the EBP, e.g., principals, administrators, teachers and students; and (e) the implementation processes that surround the EBP, including the implementation strategies used to support PGC (Damschroder et al., 2022). Although prior research has used the CFIR to identify facilitators and barriers to the implementation of school-based mental health interventions and programs (Crane et al., 2021; Eisman, Palinkas et al., 2022; Huang et al., 2014), the CFIR model has not yet been applied to analyze the implementation of a school-based SEL program in rural settings in mainland China. In general, little research has examined the barriers and facilitators that dual-role rural teachers face when implementing SEL programs, a major gap in the extant literature given the growing demand on dual-role teachers to meet students’ educational and SEL needs.

Major domains explored in the protocol included the (a) perceived evidence-base and relative advantage of the PGC, (b) the adaptability, complexity, and design of the intervention, (c) local attitudes, policies and laws regarding SEL, (d) teacher motivation and support for addressing students’ SEL and MEB well-being, (e) the relational connections in schools and the tension for change, and (f) perspectives on the resources and strategies used to support PGC, including the incentive systems (See Appendix A: Interview Guide). These specific CFIR domains were prioritized based on prior research examining SEL programs in the U.S. (e.g., Collie et al., 2015; Eisman et al., 2022; Lendrum et al., 2013; Ransford et al., 2009) and China (e.g., Ee & Cheng, 2013; Fu & Zhu, 2020; Tang, 2020; Zong et al., 2024), as well as the team’s local knowledge of the contextual factors likely to influence program uptake and sustainability in rural Chinese schools. The interview questions for each domain were derived from http://www.CFIRguide.org, a website maintained by CFIR Research Team at the Center for Clinical Management Research (2025), with questions tailored for the Positive Growth Curriculum.

Interviews were conducted in Mandarin through Wechat calls and each interview lasted about 1.5 h. All interviews were audio recorded and transcribed. After being checked for accuracy, the transcripts were translated from Mandarin into English by the first two authors, who are native Mandarin speakers. This translation was to facilitate collaboration among the research team and ensure consistency in analysis. Each implementation teacher or administrator received a 100 Chinese yuan (equivalent to $16 U.S. dollars) incentive for participating in the interview. The compensation of 100 RMB for the 1.5-hour interview was determined based on standard hourly rates within the local context. This amount was considered fair and appropriate, aligning with typical compensation practices for research participation in rural China. It reflects the average hourly wage for rural teachers, ensuring their time is valued while remaining within the study’s budget constraints. All interview procedures were approved by the University of Chicago Institutional Review Board (IRB 22-1082).

#### Positionality and Reflexivity

The three authors are all trained social workers (two doctoral candidates, one mid-career faculty member) with expertise on developing and evaluating interventions designed to improve the health and mental health of young people. The first author, born and raised in rural China, has built long standing relationships with local rural schools and teachers. As an external advisor to the RICI Foundation, she contributed to the development and evaluation of the PGC curriculum and collaborated with the Foundation to enhance the implementation of the program. She conducted the interviews in the local dialect, creating a culturally sensitive and comfortable environment for participants to share their experiences. The second author, also born and raised in China, has extensive work and research experiences in working with young children, educators, and parents within school and community settings across different cultural contexts. Specifically, he has worked in designing and evaluating SEL-related curriculum for school-aged children and teachers living in rural areas of mainland China. Like the first author, he also helped to develop the PGC curriculum and had relationships with teachers and administrators who participated in the program. The third author is a social work scholar with expertise in intervention research and implementation science. Born, raised, and educated within the U.S., the third author did not have local contextual knowledge of rural youth, communities and schools in China and was not involved in the development or implementation of PGC. In the present study, the first two authors had deep contextual and cultural knowledge regarding child and family life in rural settings, including the complex dynamics that schools and teachers face as they balance their educational commitments with nascent demands to meet student’s MEB needs. This knowledge enabled nuanced probing during the interviews and a deeper understanding of data analysis and interpretation. The third author provided scientific guidance on using the CFIR to qualitatively explore implementation, conducting Rapid Qualitative Analysis, and organizing and interpreting study findings in light of extant implementation research. Throughout the data analysis and interpretation process, the team blended the local expertise of the first two authors to situate identified determinants within their appropriate milieu. This process necessitated ongoing dialogue between the three authors. Moreover, the trust the first author established with teachers during the interviews allowed for effective member checking, enhancing the credibility of the findings.

#### Data Analysis and Interpretation

The first two authors independently coded each transcript using Rapid Qualitative Analysis (RQA), a robust, team-based approach to analyzing qualitative implementation data in order to produce actionable insights and outcomes (Hamilton, 2013). To start, the coders reviewed a selection of transcripts and developed a structured template that followed the qualitative intervention guides and CFIR constructs (Damschroder et al., 2022). The coders then independently applied the template to all transcripts before meeting to review and discuss key findings. Based on consensus, the two coders developed an analytic matrix that synthesized the key findings from each template. The coders then independently analyzed each transcript with the matrix, mapping interview text to the relevant CFIR construct(s). In addition to applying the deductively identified codes, the coders used inductive coding to capture any findings not already included in the matrix. Upon completing the final analysis, the three authors met to review the findings and to identify how CFIR constructs (Damschroder et al., 2022) operated as barriers or facilitators to shape the implementation process and key implementation outcomes (Proctor et al., 2011). The decision to categorize constructs as barriers and facilitators was based on prior CFIR coding guidance (CFIR Research Team, 2025) and a close reading of the coded data. As the three authors organized the data into barriers and facilitators, they engaged in a dialogic process about emerging themes regarding how teachers perceived the acceptability, appropriateness, feasibility and sustainability of PGC. They then returned to the matrix and interview data to organize the barriers and facilitators into four major themes, following prior guidance on the CFIR and implementation outcomes (Damschroder et al., 2022; Proctor et al., 2011). To enhance credibility of the findings, we conducted member checking by sharing the final matrix that identified the contextual barriers and facilitators with quotes with the teachers and administrators who participated in the interviews. There were no major disagreements though teachers and administrators did ask questions confirming that their responses would be anonymous. This step allowed us to confirm our interpretation of CFIR determinants with participants and to reassure them regarding the confidentiality of their responses, thereby ensuring that their voices were accurately represented and reducing the risk of researcher bias (Creswell & Miller, 2000).

## Results

We identified four major themes detailing how determinants in each CFIR domain shaped key implementation outcomes. The first two themes report the primary facilitators that contributed to high levels of acceptability, appropriateness and fidelity, while the third and fourth themes describe the contextual barriers that threaten feasibility and sustainability. Facilitators of PGC appropriateness, acceptability and fidelity included innovation characteristics, such as a high relative advantage and design quality, low complexity, and a strong perceived evidence-base (Theme 1) and the range of implementation strategies that supported teachers to deliver PGC with fidelity, especially with respect to having pre-implementation training, supportive school champions and leadership, and real-time technical assistance and facilitation (Theme 2). In addition, the results pointed to key barriers in the inner and outer setting that affected feasibility and the long-term sustainability of PGC. In the inner setting, barriers included the higher relative priority placed on academic versus SEL training and the administrative burdens and resource gaps experienced by several teachers, especially those with responsibilities beyond teaching (Theme 3). In the outer setting, the primary barriers that shaped implementation and could threaten sustainability moving forward was the lack of designated governmental resources in alignment with policies and the lack of community buy-in for SEL (Theme 4). Summary of themes and subthemes are listed in Table 3. Each theme, with relevant quotes, is described in detail below.

**Table 3:** Summary of themes and subthemes

Themes	Main Subthemes (Barriers and Facilitators)
	*Perceived facilitators to the implementation of PGC*
1: High relative advantage and design quality, low complexity, and a strong perceived evidence-base supported perceived acceptability and appropriateness of PGC	Strong relative advantage due to high unmet SEL needs Low complexity of PGC aided preparation and instruction Multimodal curriculum design supports facilitation and adaptation Belief in the effectiveness of the PGC in delivering positive outcomes PGC modifications needed to enhance developmental appropriateness (barrier and future area of adaptation)
2: Training, facilitation, and supportive leadership as core implementation strategies that supported PGC implementation	Importance of pre-implementation training for supporting teacher knowledge and self-efficacy to implement PGC Benefits of receiving multi-component facilitation strategies throughout PGC implementation Appreciated engagement from school leaders during PGC implementation Advantages of financial incentives and professional development opportunities
	*Perceived barriers to the implementation of PGC*
3: Inner setting barriers that hindered the feasibility and potential sustainability of teacher implementation of PGC	Student’s academic success has a higher relative priority than their SEL/MEB needs Difficult to manage daily competing teaching and administrative demands Challenges in delivering PGC program in a large-size class Concern and uncertainty over the ideal PGC implementer
4: Outer setting barriers that shaped feasibility and long-term sustainability	Dearth of designated financial resources aligned with governmental policy to promote student mental health Lack of local community awareness and buy-in on the importance of supporting student’s SEL/MEB health

*High relative advantage and design quality, low complexity, and a strong perceived evidence-base supported perceived acceptability and appropriateness of PGC.* Across interviews, teachers and participating administrators reported high acceptability and appropriateness of the PGC. Importantly, perspectives on acceptability and appropriateness tended to align with Proctor et al.’s (2011) implementation outcomes framework, which considers satisfaction with different innovation characteristics, including the relative advantage of the PGC, and the curriculum’s content, complexity, usefulness and perceived fit.

Teachers reported that PGC had a strong relative advantage due to high unmet mental health and SEL needs among rural students, especially the children of work-away parents. For example, one teacher (ID 8) shared her observations about her students’ struggles and the support they need to succeed: “From what I’ve observed, children born and raised in rural areas typically have lower self-esteem and confidence compared to children in urban areas. They need more resilience education to help them navigate life’s adversities and difficulties.” Other educators expressed similar beliefs that rural children needed more support than their urban counterparts. Moreover, teachers reported that these needs are greater for the children of work-away parents and children whose parents are divorced, as they “need education or skills to help them cope with these challenging circumstances” (Teacher, ID 12).

With many teachers recognizing that children had unmet SEL needs, the PGC had a high relative advantage, as nearly all interviewees (13 of 15) noted the absence of any other mental health prevention programs or services for rural students and rural areas. As one teacher (ID 15) stated, “No other mental health education program or curriculum has ever been formally implemented in my school, so the PGC fills a critical gap in our current services.” Two teachers also reported that PGC was more advantageous than a mental health education curriculum previously developed by a handful of school teachers who were interested in mental health. As one teacher (ID 2) involved in developing that curriculum stated:

Currently, there are no other programs implemented in our school. Although we developed our own mental health curriculum textbook, we never used it because we feel that our knowledge in this field is quite limited, and the curriculum we developed is not of sufficient quality to use directly.

Another teacher (ID 4) commented, “The PGC curriculum is significantly more professional compared to what we had previously developed in our mental health teacher group.”

Another innovation characteristic that shaped the high acceptability of the program was the fairly low complexity of the curriculum. Two teachers stated that implementing PGC “was straightforward and not overly complex” (ID 4 & 5). Of the 15 interviewees, 14 described how much they appreciated the user-friendliness of the curriculum, particularly the detailed instructions in the teacher’s handbook. As one teacher (ID 6) shared, the detailed instructions saved her from extensive preparations before and during class instruction:

Overall, I found no challenge in teaching this SEL curriculum. The teacher’s textbook offers clear, detailed instructions. I could easily follow them, facilitating smooth class conduct. The minimal need for prior class preparation was the greatest advantage of teaching this curriculum.

In addition to the low complexity, several interviewees appreciated the multimodal curriculum design (i.e., design quality), which facilitated in-class instruction and student engagement. Beyond a traditional textbook format filled with plain instructions for teachers, the curriculum incorporated slides, videos, cartoons, and group activities. Nine of 15 teachers and administrators also described their appreciation for the virtual component. For example, one teacher (ID 5), who taught a large class of 56 students, shared:

I found that students particularly enjoyed the videos and PowerPoint presentations. It isn’t sufficient for me to lecture for the entire class duration. The virtual component is exceptionally beneficial. On one hand, it captures students’ attention and engages them swiftly. On the other hand, it reduces my course preparation and teaching time. The textbook is also colorful and of high quality.

While the SEL textbook provided detailed instructions for teachers to deliver the curriculum, it also offered room for flexibility, allowing them to adapt specific content to their needs. For instance, one teacher (ID 7) discussed how she expanded on the activities in one of the sessions titled Controlling My Emotions in the PGC:

The final activity of this session required students to draw and write in their textbooks. Students were tasked with jotting down three emotional regulation strategies under an ‘emotional balloon’ to send as a gift back to the fire dragon, a character from our SEL curriculum cartoons. However, I took it a step further and asked my students to draw and write three strategies for themselves including methods to deal with specific emotions, such as a ‘depressed’ balloon, an ‘angry’ balloon, and an ‘anxious’ balloon. My students found these additional activities to be particularly practical.

Several interviewees (6 out of 15) also reported how their belief in the effectiveness of the SEL curriculum increased after observing student changes after delivery a few sessions. For example, one teacher (ID 11) recounted observing a positive change in one of the students enhanced her acceptance of the PGC:

I had a student who previously struggled with significant emotional issues. He told me he found the concepts and strategies he learned in class to be extremely beneficial, so much so that he no longer becomes easily irritated by minor incidents. He can better control his emotions now. This experience made me trust and appreciate the program even more.

Despite the overall positive views of the curriculum, 6 out of 15 interviewees observed that the curriculum needed additional tailoring to be developmentally appropriate, as some of the games and group activities were overly simplistic for fifth graders. They suggested modifying these activities to be more age-appropriate, especially given the influence of digital devices on children today. One teacher (ID 3) offered suggestions on how to improve PGC to meet students’ needs:

Fifth graders nowadays are more mature than they were a few years ago, partly due to the significant impact of digital devices on rural children. They have a broader knowledge base than their peers did five or 10 years ago. Incorporating more complex activities that better fit their needs or preferences would enhance the implementation of the SEL curriculum. There’s a need for additional development in the SEL curriculum itself to tailor it more effectively to the needs of fifth graders.

*Training*,* facilitation and supportive leadership as core implementation strategies that supported PGC implementation.* Teachers had overall positive attitudes towards the implementation strategies, though several offered suggestions for improving the types of strategies that could facilitate and sustain teacher implementation of the PGC within their school. Given that most teachers were unfamiliar with SEL-related concepts, almost every teacher had positive feedback on the training, which covered basic information regarding SEL, child mental health issues, and specific instructions on implementing the PGC. For example, one teacher (ID 6) described how the day-long training improved her confidence in teaching the curriculum on her own:“I feel much relieved after the training and feel I am capable of starting to teach a SEL class I have never experienced before.” Another teacher (ID 13) echoed the sentiment that the training improved their self-efficacy to teach the curriculum, especially as someone who was not a trained mental health professional:

I am a regular school math teacher, not a trained mental health professional, so I was quite scared and worried about delivering the PGC curriculum on my own, as I had no prior experience teaching such a curriculum. The training was extremely helpful because the staff not only showcased sample classes but also involved us in pilot teaching. After I piloted one course session, both the participating teachers and trainers provided very positive feedback. They encouraged and complimented me, while also offering constructive comments for improvement. I feel much more confident in teaching the entire curriculum package on my own after the training.

In addition, several teachers expressed how the condensed training format, as opposed to a longer week-long course, helped them to manage their tasks and obligations in their daily lives. For example, one teacher said “the full-day intensive training was very helpful and didn’t take too much of our time. I really like this format!” Her thoughts were shared by another teacher (ID 10), who pointed out that “having a short period of in-person training, like one or two days, is perfect for me.” However, three teachers reported that future training iterations should use virtual training sessions, as this would better accommodate their demanding school schedules.

In addition to the importance of training, teachers described several benefits of the multi-component facilitation strategy, including the online learning collaborative hosted on WeChat. In their responses, teachers appreciated having access to expert facilitators who could help them to troubleshoot emergent issues, and learning from other teachers who were delivering PGC. As one teacher (ID 14) indicated:

I really like the WeChat group! Whenever I had questions about implementing the PGC program, the program staff and curriculum developers responded promptly. Sometimes, other teachers who encountered the same issue or had a solution also shared their thoughts in the chat group. We, as participating teachers, often shared our experiences—both good and not so good—of teaching the PGC curriculum. The other members of the group were very supportive, offering feedback and advice. It was a great learning community!

Another teacher (ID 2) echoed this point, stating:

The WeChat group has created a community for us to learn and solve problems together. I recall helping another teacher solve an issue regarding the technology needed to display the slides and videos for the course. Moreover, in the group, I not only asked questions relevant to teaching the SEL session, but I also shared my experiences and thoughts on how to support students’ well-being in general with my colleagues.

Teachers also expressed their strong appreciation for having high level leaders, such as principals and the dean of *deyu*, champion the program, as their leadership was viewed as central to successfully implementing PGC in their schools. For example, one teacher (ID 13) reflected on how pivotal it was to have the principal’s support during implementation. She stated, “The school principal is crucial. Only if they support the curriculum, is there a chance that a new curriculum [such as PGC] can be implemented.” Her points were supported by an administrator (ID 9), who added: “The support of the school principal is also crucial for continuing this kind of new program in the long run.”

In addition to highlighting the importance of school leaders’ engagement in the PGC, several teachers emphasized their appreciation for their leaders’ approachability during implementation, underscoring the role of the individual characteristics domain (Damschroder et al., 2022). This sentiment was illustrated in the response from ID 3, a teacher who expressed her appreciation for the dean of deyu’s approach to supporting PGC implementation:

[Name redacted] provided excellent support for the implementation of the PGC program at our school. The dean of deyu conducted regular check-ins to monitor our progress and offered assistance whenever needed. Although they hold a leadership position, the dean didn’t use their authority to pressure us into teaching the class on a strict schedule. Instead, they took a friendly, easygoing approach, encouraging us to implement the program at our own pace. Their warm words and strong relationships with nearly all the teachers made them a beloved figure in our school.

Teachers’ perceptions of this leadership style were echoed by both champions and principals, who described adopting a leadership style designed to cultivate rapport and trust among teachers delivering PGC. For example, the dean of deyu in one school (ID 15) reflected upon the entire process and their rationale for pursuing a collaborative leadership style:

I made an effort to leverage my personal connections with each participating teacher to help them stay on track with their teaching progress. Instead of formal check-ins, I opted for a more informal approach, such as discussing progress during lunch hours. It was not my intent to use my leadership role to force compliance, but rather to use my personal relationships with teachers. I found this approach to be more effective in maintaining progress.

Finally, most teachers were grateful for the financial incentives and viewed the incentive as an important strategy for helping to cope with the barriers associated with taking on additional work. For instance, one teacher (ID 12) mentioned that “the cash incentives were a pleasant surprise, making me feel more appreciated for the extra time invested in teaching each SEL session.” Another teacher (ID 1) noted that “additional cash support was particularly beneficial for our rural teachers, who typically earn much less than our urban counterparts.” Regarding the appropriate amount, some teachers suggested that a rate of 100 to 200 RMB per session ($13 to $27 U.S. dollars) would be sufficient to motivate them to continue implementing similar programs in their schools. One teacher, however, disagreed with the need for financial payments and offered an alternative incentive format: “I’m not particularly interested in cash incentives. If there were opportunities for continuous professional development, especially through virtual platforms, I would feel more motivated by that type of incentive.”

*Inner setting barriers that hindered the feasibility and potential sustainability of teacher implementation of PGC.* Of particular note, all 15 interviewees described how delivering SEL programming was a lower relative priority than supporting students’ academic achievement. Teachers felt this pressure keenly, which they identified as manifesting in both the inner school setting and outer community and policy contexts (See Theme 4 for Outer Setting barriers). For example, one teacher (ID 6) stated: “Implementing the PGC curriculum was not a priority for our school compared to regular classes.” One administrator (ID 9) elaborated:

Both teachers’ and the school’s performance reviews are primarily based on student academic outcomes, according to current educational policies. As a result, schools are compelled to prioritize regular classes over those that they think do not contribute to students’ academic records, such as this kind of SEL program.

In addition to their teaching load, teachers in rural schools are often expected to have other responsibilities, especially demands to take on more administrative roles within the school. For example, in China, the banzhuren is a teacher who is “the leader of teachers and the key person responsible for developing the whole community of students” (Gu et al., 2015, p. 102). In this role, the banzhuren not only teaches the same community of students across several years but also communicates with and develops leadership among their fellow teachers (see Gu et al., 2015). All 13 teachers, including those serving as a banzhuren, discussed the difficulties of managing their daily administrative work (Inner Setting: Structural Characteristics, Work Infrastructure and Culture). As a result, many found that adding an SEL curriculum further compounded this burden. ID 13, a teacher who worked as the banzhuren described the competing demands they experienced:

The administrative burden is overwhelming. I spend about 30% of my time teaching and the remaining 70% on unnecessary administrative tasks. Especially as the banzhuren, I am responsible for safety education, law education, sometimes even physical education classes, and other school activities.

Another teacher (ID 10) shared her daily stress in dealing with administrative tasks, saying:

I feel overwhelmed by the current workload, especially the endless administrative work, like filling out unnecessary forms for inspections from the central city government. Teaching the additional PGC program was not easy for me; I had to find extra time to do it.

In addition to the heavy administrative burdens teachers reported experiencing as part of their routine work in schools, 10 out of 13 teachers reported that large class sizes negatively impacted the quality of SEL curriculum delivery. Indeed, the average class size among the 13 classrooms participating in the SEL intervention was around 50 students. One teacher (ID 2) shared her struggles with teaching SEL content to such a large group of students:

My class size is too large, often exceeding 50 students. The classroom isn’t spacious enough to accommodate group activities effectively. A smaller class size would significantly improve the learning experience for students. As a teacher, a smaller class would also alleviate some of the burdens associated with managing such a large group.

These barriers led some teachers to express uncertainty over whether teachers should be the ideal implementers for PGC moving forward. While some teachers suggested the need for a dedicated full-time teacher or staff member to deliver the SEL curriculum, others argued that a more pragmatic approach would involve appointing a teacher to take on extra responsibilities, given that there were no provisions for recruiting full-time staff. Particularly, they suggested that the ‘banzhuren,’ who generally has a comprehensive understanding of each student in the class, would be the optimal choice over those discipline teachers who were responsible only for teaching non-math or Chinese courses. One teacher (ID 10) shared her dilemmas on appointing ‘banzhuren’ as the lead teacher in instructing the SEL curriculum:

The ideal scenario is having a ‘banzhuren’ who is passionate about teaching this in their own classroom. They have an advantage in that they know every student well and can manage the classroom with ease. However, the prerequisite for this would be to relieve their already heavy administrative burden or provide additional support such as cash incentives or professional development opportunities.

*Outer setting barriers that shaped feasibility and long-term sustainability.* Teachers who implemented PGC identified also two outer setting barriers that affected the perceived feasibility, i.e., the program’s actual utility and suitability for daily use, and sustainability, i.e., the program’s ability to be maintained, continued, and integrated for long-term use (Proctor et al., 2011). Many teachers described the challenges of not having designated financial resources that correspond with governmental policy priorities to support student mental health. For example, an administrator (ID 9) described the dearth of mental health professionals for students in their elementary school: “Our whole county only has 32 school psychologists, all of whom are allocated to middle and high schools, not elementary schools. Only recently has the county established a mental health counseling center, which is based in the urban area.” An administrator (ID 15) in a separate school likewise stated: “although central government policies require each school to have at least one dedicated staff member or teacher responsible for student mental wellbeing, no funding has been allocated to hire such a person. Without dedicated personnel, no additional mental health programs in schools can be implemented or sustained.”

Interviewees also reported that local attitudes and beliefs regarding mental health and SEL programming in rural areas was a major outer setting barrier. Teachers and administrators shared that community members in rural areas, including parents and grandparents, were often unaware of the importance of child mental health or the value of social and emotional learning. For example, one teacher (ID 5) noted that “caregivers in rural areas paid very little attention to children’s emotional needs, focusing primarily on academic performance and showing little interest in their participation in other activities or programs.” Similarly, her observation and concern was shared by another teacher (ID 14), who mentioned that “I don’t think people in rural areas really know or care much about children’s mental health. Some of them probably care more about their child’s academic performance and do not care whether their kids are happy, angry, depressed….”

The lack of caregiver recognition and understanding of children’s mental wellbeing presented another challenge for some teachers, as they struggled to communicate with parents regarding student’s mental wellbeing and the importance of programs like PGC. One teacher recounted a particularly difficult experience talking to a parent about their child’s behavior:

I had a student who was stealing others’ belongings, so I spoke with the student’s parents. However, their response was alarming: ‘I will ask my child to jump into the river (commit suicide).’ As a teacher, I am now afraid to have such conversations with parents who seem to know and care little about their children’s mental or emotional well-being. They are only concerned about their children’s grades.

## Discussion

Teacher-led SEL programs to support student mental health are a promising intervention approach, especially in contexts like rural China, where there is a limited mental health infrastructure and few trained mental health professionals (Wu & Pan, 2019). This qualitative study explored the facilitators and barriers experienced by teachers and administrators implementing the Positive Growth Curriculum, an efficacious teacher-led SEL program that was implemented in two rural elementary schools in southwest China (Fu et al., 2024). Both SEL and mental health programming in China is growing, with focused efforts to improve the mental well-being of the children of work-away parents, who face unique stressors due to their parent’s economic migration (Wang et al., 2019). To date, little research has identified the contextual determinants that shape the implementation of teacher-led SEL programs for rural children, including the factors that may affect their sustainability. Our results point to several facilitators and barriers that must be harnessed and addressed to bring PGC to scale, which may support ongoing efforts to task-shift mental health programming to teachers (Fazel et al., 2014; Vanderburg et al., 2022b).

Of particular note, one of the primary facilitators of PGC was the high relative advantage of the curriculum. Numerous teachers noted that the PGC program addressed an important gap, as there were few available programs designed to support the mental health of rural children. Consistent with research on the prevalence of MEB challenges among rural children (Chen et al., 2019), teachers in our study perceived that MEB challenges were more common among the children of work-away parents. Given these needs and the policy mandate that schools address student mental health, our results indicate that PGC provided teachers with an appealing alternative. Even teachers who had attempted to develop their own SEL program indicated that PGC had a stronger relative advantage. To date, few studies in China have examined the role of relative advantage in supporting the implementation of a teacher-led SEL program–a gap identified in other research on the determinants of school-based mental health programs (see Richter et al., 2022). However, as Greenhalgh et al. (2004) note, relative advantage is considered “sine qua non” i.e., essential, for adoption (p. 594), which our results also support.

In addition to the strong relative advantage, we found that the low complexity and high design quality and packaging of PGC were important facilitators of teacher acceptability and appropriateness. Almost all interviewees described how easy it was to follow the clear and detailed instructions in the textbook during implementation. In a recent review of contextual determinants that shape the implementation of school-based mental health programs, design quality and packaging was the most commonly studied dimension in the innovation characteristics domain of the CFIR (see Richter et al., 2022), potentially reflecting the ease of assessing this construct. However, prior research on SEL programs has stressed the importance of design quality and packaging for supporting implementation (e.g., Gallagher et al., 2023; Leeman et al., 2018; Lendrum et al., 2013; Richter et al., 2022), as well-designed materials help to reduce the complexity of implementing a new program and give important roadmaps to teachers, many of whom are juggling multiple responsibilities.

Despite identifying strong facilitators in the innovation characteristics domain of the CFIR (Damschroder et al., 2022), teachers also identified a number of key barriers. With respect to the adaptability of the curriculum, several interviewees reported struggling with a lack of age-specific content and activities. Although the development team from the RICI Foundation piloted the curriculum with rural teachers, children were not involved in developing the program. Moving forward, intervention developers can partner with teachers, administrators, and rural children and their families, including the children of work-away parents, to adapt the curriculum and develop developmentally appropriate content. This approach is consistent with calls for more community engagement in intervention development (Stover et al., 2024), including the development of culturally appropriate community-based participatory research methods in China (Liu et al., 2011).

Community engagement also could address the outer setting barrier of low community awareness of the importance of mental health, as teachers reported that families placed a lower relative priority on student mental health versus academic learning. Prior research on scaling effective SEL programs in diverse communities has underscored the importance of creating community coalitions to disseminate information on the effectiveness of SEL programs, which can enhance community buy-in (Fagan et al., 2015). Although not implemented as part of PGC, multisystemic partnerships between schools, families, and community stakeholders are a core feature of the CASEL framework (Borrowski, 2019). In addition, prior research examining task-shifting mental health care to teachers has found that family engagement is a major perceived facilitator (Vanderburg et al., 2022). Moving forward, an approach that engages parents and families may be helpful for implementing and sustaining teacher-led SEL programs in rural communities in China, given the relatively low awareness of mental health issues and high rates of stigma towards mental illness (Huang et al., 2019; Yin et al., 2020). Community coalitions also could help to develop campaigns that highlight the ways in which SEL supports students’ academic achievement, thereby capitalizing on the high value placed on education and framing SEL as part of teacher’s roles and responsibilities in supporting children’s academic success.

While teachers recognized that recent policy efforts from local and national governments are beneficial for implementing SEL programs like PGC, they also expressed concerns over a kind of policy tokenism, wherein teachers and schools were mandated to provide mental health programming without being given the resources and support necessary to address this complex challenge. Rural teachers in China experience low job satisfaction and high rates of burnout and attrition, which have worsened since the pandemic (Wang et al., 2022). In China, rural teachers face additional tasks such as poverty alleviation, family visits, individual support for children with intellectual disabilities, and other responsibilities (Fu & Zhu, 2020). In this study, interviewees discussed the challenges of balancing their educational, administrative, and other responsibilities with offering PGC, a key barrier to future implementation and sustainability. For some teachers, this tension was reflected in comments that PGC implementation was a lower relative priority in their school. These challenges also were compounded for teachers like the banzhuren, who already carry a high administrative workload. For other teachers, the high workload led to some uncertainty about who the ideal implementer should be, with some teachers suggesting banzhuren fulfill this role. Banzhuren teachers, in turn, were clear that they needed relief from their administrative duties in order to support this work. Thus, additional work is needed to support teachers to implement effective programs like PGC without overburdening them, as teacher burnout and attrition not only threatens the sustainability of EBPs like PGC (see Pascoe et al., 2021 for a review), but also negatively affects student academic achievement (Ronfeldt et al., 2013).

Teachers also noted that factors such as teaching a large class size and having small classrooms—two features of the inner setting shaped by access to outer setting resources—made implementation challenging. It remains unclear, however, how challenges associated with external financial constraints and school structural characteristics could be adjusted and balanced to achieve more successful implementation. One potential solution could be to implement PGC as a targeted rather than as a universal prevention program. In the recent evaluation of PGC (Fu et al., 2024), the effects on SEL outcomes were more pronounced for the children of work-away parents. However, teachers and schools may not have the additional capacity, resources, or space to mount a specialized curriculum for children of work-away parents, which could require teachers and schools to screen children for eligibility and require additional space and resources; separating identified children from their peers also could increase stigma among the children of work-away parents, as universal interventions may be less stigmatizing (Stallard, 2013). This barrier will need particular attention for PGC and other task-shifting efforts that seek to leverage teachers to support student mental health in China.

Implementation strategies are central for supporting the adoption of any new innovation (Powell et al., 2015), and play an important role in helping teachers to integrate evidence-based interventions into their work with students (Moore et al., 2024). Our results indicate that teachers had overall favorable views of the implementation strategies deployed by the RICI Foundation, which included training, facilitation and active involvement of school leaders and champions. As in much of the implementation research, including those focused on school-based SEL programs (e.g., Reinke et al., 2011), training and facilitation were considered foundational strategies (Edmunds et al., 2013) to support teachers to implement PGC with fidelity. Teachers noted that training improved their own knowledge and self-efficacy to implement PGC, and appreciated the flexibility and convenience of the brief training session. As many teachers lacked knowledge of SEL, the training also may have helped to reduce the complexity of implementing PGC. The knowledge and skills gained in the training were further supported by the online learning collaborative, which provided teachers with an opportunity to connect with their peers and RICI staff in order to problem-solve emergent issues in real time. Learning collaboratives are a commonly used strategy to support mental health providers to deliver evidence-based interventions (for a review, see Gotham et al., 2023), and are effective for supporting student-based mental health programs (Orenstein et al., 2023), though less research has examined their utility and effectiveness among rural teachers in China.

In this study, teachers noted that having trustworthy leaders who championed the program was vital to their ability to successfully implement PGC. This sentiment was echoed by administrators, who emphasized that the informal trust and connections they established with teachers was critical for supporting implementation, including holding participating teachers accountable in terms of quality implementation of the SEL program. A growing body of research has underscored the importance of trustworthiness and psychological safety in successful implementation efforts (Byeon et al., 2022; Fleming et al., 2023), and studies suggest that school leaders play an important role in facilitating a positive implementation climate (Collie et al., 2015). Given the salient roles of principals and other administrative leaders in Chinese educational systems, support from rural school leaders is likely a key facilitator for task-shifting mental health programs like PGC to teachers. Future SEL implementation should continue incorporating strategies to facilitate relational trust between teachers and administrators. Thus, ensuring top-down policies align with teachers’ practical capacities and needs in implementing mental health education within rural school settings remains a significant challenge.

In recent years, China has passed several policies and programs designed to increase economic investments in rural education (Li & Liu, 2014; Wang et al., 2019). One such policy, the Rural Teachers Support Plan, has increased salaries for teachers in rural areas (Ministry of Education, 2016), with some evidence indicating that increased pay for teachers has significantly improved student test scores (Liu et al., 2022). In line with these efforts, the RICI Foundation provided teachers with a modest financial incentive after completion of each SEL session (100 RMB/$13 USD per session for a total of 800 RMB/$110 USD). Teachers appreciated this incentive, noting that it helped to offset the increased workload associated with implementation. Although financial incentives have been used to support therapists and other formally trained mental health providers to deliver interventions (Garner et al., 2018; Paris & Hoge, 2010), the effectiveness of incentives for teacher-led SEL programs is less clear (for a review, see Moore et al., 2024). As the incentive funds in the current study were provided by an external funder, schools implementing PGC moving forward may not have access to these resources. Of interest, one teacher noted a preference for incentives in the form of professional development opportunities. Given that China requires teachers to pursue professional development to maintain their teaching credentials (Han, 2012), professional development opportunities could be one way to incentivize teachers. Future research should partner with teachers and schools to explore feasible, impactful and sustainable incentive structures.

## Limitations, Strengths, and Future Directions

While these findings yield important insights into the contextual determinants that shaped the implementation of PGC, a teacher-led SEL program for rural children in China (Fu et al., 2024), study findings should be interpreted in the context of the limitations. First, interviews were conducted during the summer, one month after PGC implementation had concluded. Although the retrospective approach helped to reduce the demands placed on teachers, teacher reports could be subject to recall bias. Future research should examine implementation in multiple stages, including pre-implementation and throughout the implementation process, which will provide real-time data on the contextual factors that shape implementation over time. Ethnographic research that includes participant observation of teachers and students, as well as interactions between administrators and teachers, could yield additional on-the-ground insights to drive implementation strategy updates or to capture adaptations that teachers make in response to student needs (Gertner et al., 2021). Another limitation of this study is its small sample size, which included only 13 teachers and two administrators from two rural elementary schools. Our analysis did not reveal any major conflicting viewpoints between teachers and administrators. Administrators tended to emphasize broader factors affecting implementation—such as feasibility, sustainability, and long-term integration—while teachers focused on day-to-day challenges and practical aspects of classroom implementation. These findings highlight the need to follow current CFIR guidance to the different roles of those associated with implementing an EBP (Damschroder et al., 2022). Future research with a larger and more diverse sample may help to uncover more nuanced perspectives on implementing SEL programs in rural school settings, including similarities and differences between teachers, principals and other key stakeholders. Considering the significant variation among rural schools in China, our findings may not fully capture the diverse contexts in which SEL programs are implemented. While this study generates rich information regarding how the agency and school districts can better prepare for the future implementation of teacher-led SEL programs, study findings may not generalize to urban or other rural contexts. Future research should explore SEL implementation across a broader range of rural schools to better understand the factors that contribute to successful program adoption in different regional contexts.

To the best of our knowledge, this is the first study to examine the barriers and facilitators to implementing a school-based mental health prevention program in rural Chinese schools. To date, most implementation science research in China has focused on healthcare settings, with less attention to other settings where evidence-based programs could offer promise for improving health outcomes. Although some prior work has examined task-shifting mental health programming to teachers, this research has focused on university faculty and not rural elementary school teachers (Hou et al., 2024). As demonstrated in existing evidence, many existing youth mental health prevention programs in China have largely overlooked the identification and exploration of potential barriers and challenges encountered throughout the implementation process (Yang et al., 2025). As such, the findings from this qualitative study fill an important knowledge gap on the implementation of teacher-led SEL programs in rural China and have several implications. First, additional research is needed to build the research base on the contextual determinants that shape the implementation of teacher-led SEL programs in rural areas, as there may be important differences in important CFIR domains, including school infrastructure and implementation climate (inner setting), personnel competencies (individual factors), and community buy-in (outer setting) between different rural schools (Damschroder et al., 2022). As such, different schools may need different types of implementation strategies or strategies may need to assume different forms in order to address local contextual needs (Perez-Jolles et al., 2019). Additional research on the implementation determinants, strategies, and outcomes involving various types of SEL-related programs will yield significant insights for program developers, researchers, and practitioners interested in task-shifting mental health programs to teachers in order to support children’s social and emotional mental wellbeing across mainland China. As our results indicate, and as studies in and outside of China have documented (Liu & Onwuegbuzie, 2012), teacher workload remains a significant barrier to having teachers deliver evidence-based programs. Teachers in rural schools in mainland China face extra barriers and challenges associated with societal expectations, class structures, and job responsibilities to the effective implementation of SEL-related interventions, which may increase burnout and turnover (Liu & Onwuegbuzie, 2012). Therefore, culturally sensitive strategies to address these factors must be taken into consideration. This work will likely require additional efforts to ensure that schools and teachers have adequate resources to address the policy mandate of supporting student mental health. Importantly, all teachers evidenced high motivation for supporting the SEL and mental health of their students, especially the children of work-away parents. This motivation indicates that it is possible to partner with teachers to support student mental health. Indeed, our study underscores the perceived benefits of several implementation strategies used to support teachers. Future research should utilize and expand these strategies to better promote the effective implementation of teacher-led SEL programs.

## Conclusion

The Positive Growth Curriculum was delivered by teachers to over 600 rural fifth grade students in China (Fu et al., 2024). It and other teacher-led SEL programs offer significant promise for meeting the mental health needs of rural children in China, especially the children of work-away parents. This research sheds light on the importance of understanding the implementation process of school-based SEL programs such as PGC and calls for more efforts to study, sustain and scale such programs up in order to support teachers to advance children’s mental health.

## Electronic Supplementary Material

Below is the link to the electronic supplementary material.

Supplementary Material 1
