# Supplementary material for: Barriers and Facilitators to Implementing a School-Based Social and Emotional Learning Program for Rural Children in China: A Qualitative Study Using the Consolidated Framework for Implementation Research
Source: Adm Policy Ment Health. 2025 Jul 15;52(6):1199–218. doi: 10.1007/s10488-025-01460-z (PMC12628482; doi:10.1007/s10488-025-01460-z)
Supplement: Supplementary file 1 — Supplementary Material 1 [file 10488_2025_1460_MOESM1_ESM.docx]

**Interview Guide with Teachers/Administrative Staff Based on CFIR 2.0**

**访谈提纲（教师/行政人员）基于CFIR2.0框架**

Thank you so much for your willingness to participate in our study. We want to learn more about your experience with the intervention and what’s been working or not working for you. As a reminder, there are no right or wrong answers to the questions; we value your perspective in helping deliver the SEL intervention. We will keep your responses confidential.

非常感谢您愿意参与我们的研究。我们希望通过此次访谈去进一步解您在实施本项目时的经验及感受。在本次访谈中，您对于任何问题的回答都没有对错之分。我们十分重视您分享实施本次社会情感学习(SEL)干预的经验及反馈。所有本次访谈内容均会严格保密。

**(Permission to record) 询问是否可以录音**

**Social demographic information (age, school, class size) 基本信息（年龄，学校，班级人数）**

1. **Overview of teacher’s role in school (1 question) 教师工作职责总览（1题）**

Q1. To begin our conversation, I would like to learn more about you and your role in the school and how you have been involved in implementing the SEL program this semester. What are your specific roles and responsibilities within your school? (Only working as a teacher or having an additional administrative role?)

首先，我想更多地了解您的一些信息。您在学校担任什么角色？您是如何参与到实施本学期社会情感学习项目的？

您在学校中的具体工作职责是什么？（仅担任教师或担任额外的行政职务？）

1. **SEL Intervention Characteristics (6 questions) 关于课程包的特征问题（6题）**

| **CFIR Construct** | **Teachers Who Implemented the SEL program**  **教授课程的老师** | **Administrative staff or leaders in the school**  **学校负责的行政人员** |
| --- | --- | --- |
| Innovation Domain  创新领域 | Now, we are going to discuss some of your more general impressions about implementing the SEL in your class.  现在我们来聊聊您对于教授这门课程的总体印象。 | Now, we are going to discuss some of your more general impressions about implementing the SEL in your school.  现在我们来聊聊您对于在学校实施这门课程的总体印象。 |
| Evidence-Base  循证 | To what extent do you believe that the school-based SEL program can support students in preventing mental health disorders/challenges, reducing mental health symptoms, as well as improving academic performances?  您认为该课程在多大程度上可以促进学生的心理健康发展、减少心理健康症状以及提升他们的学习成绩？ | To what extent do you believe that the school-based SEL program can support students in preventing mental health disorders, reducing mental health symptoms, as well as improving academic performances?  您认为该课程包在多大程度上可以促进您学校学生的心理健康发展、减少心理健康症状以及提升他们的学习成绩？ |
| Relative Advantage  相对优势 | Is there another intervention that you would rather implement? If so, can you describe more about that intervention?  你们学校是否有其他类似的干预或者项目？如果有的话，是否可以请您多分享一些关于那个项目的相关信息。 | Is there another intervention that you would rather implement? If so, can you describe more about that intervention?  你们学校是否有其他类似的干预或者项目？如果有的话，是否可以请您多分享一些关于那个项目的相关信息。 |
| Adaptability  适应性 | What kinds of changes or alterations you have already made or you will need to make to the SEL intervention so it will work more effectively in your class? Are there components that should not be altered?  您是否调整了（或觉得应当调整）相应的课程内容，以便其在课堂上更有效地发挥作用？有哪些课程内容是您觉得不应该调整的？ | What kinds of changes or alterations you have already made or you will need to make to the SEL intervention so it will work more effectively in your school?  您是否调整了（或觉得应当调整）相应的课程内容，以便其在课堂上更有效地发挥作用？ |
| Complexity  复杂性 | What do you think of the complexity of the curriculum? On a scale of 1-10, with 10 being the most difficult/complicated, from you perspective, how difficult is it for you to deliver the SEL curriculum  您觉得上这个课程复杂吗？如果请您从依照1-10分的维度为其打分，10分代表最困难/最复杂的，您会打几分？  Please consider the following aspects of the intervention: duration, scope, intricacy and number of steps involved and whether the intervention reflects a clear departure from previous practices.  在您打分时，请考虑该课程的持续时间、范围、复杂性和所涉及的步骤，以及干预是否与以前做法的明显不同。 | On a scale of 1-10, with 10 being the most difficult, from your perspective, how difficult is it for you to implement the SEL intervention in your school?  您觉得在您学校实施这个课程复杂吗？如果请您从依照1-10分的维度为其打分，10分代表最困难/最复杂的，您会打几分？  Please consider the following aspects of the intervention: duration, scope, intricacy and number of steps involved and whether the intervention reflects a clear departure from previous practices.  在您打分时，请考虑该课程的持续时间、范围、复杂性和所涉及的步骤，以及干预是否与以前做法的明显不同。 |
| Design | What is your perception of the quality of the curriculum package and toolkit? Why?  您觉得该课程和工具包的总体质量如何？为什么？ | What is your perception of the quality of the curriculum package and toolkit? Why?  您觉得该课程和工具包的总体质量如何？ 为什么？ |
| Cost | What do you think about the additional costs?  您教授该课程是否涉及到任何额外的花费？ | What additional costs will be incurred to implement the intervention for your school?  您学校实施该项目是否涉及到任何额外的花费？ |

**IV. Outer Setting (3 questions) 外部环境（6题）**

| **CFIR Construct** | **Teachers Who Implemented the SEL program**  **教授课程的老师** | **Administrative staff or leaders in the school**  **学校负责的行政人员** |
| --- | --- | --- |
|  | Next, we are going to explore factors within the larger and external environment that can possibly influence (both positively and negatively) your ability to implement the SEL in your class.  接下来，我们将探索在外部环境中（社区、家庭、社会等）可能（正向或负向）影响您教授该课程的相关因素。 | Next, we are going to explore factors within the larger and external environment that can possibly influence (both positively and negatively) your ability to implement the SEL intervention in your school.  接下来，我们将探索在外部环境中（社区、家庭、社会等）可能（正向或负向）影响您在学校中实施该课程的相关因素。 |
| Local Attitudes  当地态度 | What do you think about parents’ or community’s attitudes toward teaching mental health prevention classes for your students?  您觉得学生父母或社区对于您为学生教授该课程的态度是怎样的？ | What do you think about parents’ or community’s attitudes toward opening mental health prevention classes in your school?  您觉得学生父母或社区对于您学校实施该课程的态度是怎样的？ |
| Policies & Laws | What are your impressions of how local governmental policies or national policies support or create barriers to the implementation of a SEL program in your school?  您如何看待当地政府或国家政策对于您教授课程的影响？ | What are your impressions of how local governmental policies or national policies support or create barriers to the implementation of a SEL program in your school?  您如何看待当地政府或国家政策对于您学校实施课程的影响？ |
| Financing | Is there any available fund for mental health education that your school can apply for either from the local government or other sources?  您的学校是否有来自于当地政府或其他渠道针对心理健康相关的专项资金支持？ | Is there any available fund for mental health education that your school can apply for either from the local government or other sources?  您的学校是否有来自于当地政府或其他渠道针对心理健康相关的专项资金支持？ |

**V. Inner Setting (8 questions) 内部环境（8题）**

| **CFIR Construct** | **Teachers Who Implemented the SEL program**  **教授课程的老师** | **Administrative staff or leaders in the school**  **学校负责的行政人员** |
| --- | --- | --- |
|  | Now, we are going to focus on factors at the school-level that can influence the implementation of the SEL program in your school  现在，我们将聊聊在学校内部环境中可能影响您教授该课程的相关因素。 | Now, we are going to focus on factors at the school-level that can influence the implementation of the SEL program in your school  现在，我们将聊聊在学校内部环境中可能影响您实施该课程的相关因素。 |
| Physical  Infrastructure  物理环境 | Is your classroom good for teaching the SEL class (class size, space for activities)?  您的教室是否适合教授该课程 （班级规模、活动空间）？ | Is your school good for teaching the SEL class (class size, space for activities)?  您学校的教室是否适合实施该课程 （班级规模、活动空间）？ |
| Work  Infrastructure  工作环境 | How do you feel balancing your teaching workload (other school obligations) and the new SEL teaching workload?  您如何平衡自己的日常教学工作（或其他学校职责）和教授这门新的课程？  How about introducing a dedicated staff?  您觉得专业教师是否更适合教授该课程（副科老师或者专门的心理老师） | Do you feel it added an extra layer of burden for the teachers to balance your teaching workload and the new SEL teaching workload? Are there any supports for them?  您是否觉得对于你们学校的老师增加这门课程会造成额外负担？学校是否有任何支持？  How about introducing a dedicated staff?  您觉得专业教师是否更适合教授该课程（副科老师或者专门的心理老师） |
| Relational  Connections  人际关系 | Can you describe your working relationships with your colleagues? Did you seek support from them while teaching the SEL curriculum? Are there any group meetings?  您能描述一下您与同事的工作关系吗？ 您在教授该课程时是否寻求过他们的支持？您有参加过与课程相关的小组会议吗？ | Can you describe the working relationships among teachers in fifth grade? Did they seek support from each other while teaching the SEL curriculum? Are there any group meetings?  您能描述一下五年级授课老师之间的工作关系吗？ 他们在教授该课程时是否相互支持？ 有与课程相关的小组会议吗？ |
| Tension for Change  改变的压力 | What do you think about the overall mental health and well-being of your students? What are the major problems?  您如何看待学生的整体心理健康状况？您觉得他们面临的主要问题是什么？ | What do you think about the overall mental health and well-being of students in your school? What are the major problems?  您如何看待学生的整体心理健康状况？您觉得他们面临的主要问题是什么？ |
| Relative Priority  相对重要性 | What are the high-priority initiatives or activities in your school?  Did the implementation of the SEL intervention conflict with these priorities? (Probe the double burden reduction policy and the academic performance goal)  您所在的学校哪些事情是优先级的？  该课程的实施是否与这些优先事项相冲突？ （探讨双减政策与学业成绩目标） | What are the high-priority initiatives or activities in your school? Did the implementation of the SEL intervention conflict with these priorities?  (Probe the double burden reduction policy and the academic performance goal)  您所在的学校哪些事情是优先级的？  该课程的实施是否与这些优先事项相冲突？ （探讨双减政策与学业成绩目标） |
| Incentive Systems  激励机制 | Do you think the current cash incentive (800 RMB) is good enough for you to teach the additional 8 SEL sessions? Why? Any other advice?  您认为目前对于教授该课程（8节）的现金奖励（800 元人民币）是否充足？ 为什么？ 您有什么建议吗？ | Do you think the current cash incentive (800 RMB) is good enough for you to teach the additional 8 SEL sessions? Why? Any other advice?  您认为目前对于教授该课程（8节）的现金奖励（800 元人民币）是否充足？ 为什么？ 您有什么建议吗？ |
| Mission Alignment  使命吻合 | How does providing students with the SEL curriculum fit or not fit with the mission of your school?  为学生提供该课程是否和您学校的使命相吻合？ | How does providing students with the SEL curriculum fit or not fit with the mission of your school?  为学生提供该课程是否和您学校的使命相吻合？ |
| Available Resources  可用资源 | What do you think of the training offered by Rici staff members? How helpful was the training for you to teach the class? Are there any other available resources or resources that you need?  您如何看待日慈员工所提供的课程培训？ 您觉得这些培训对您实际授课有多大帮助？ 您觉得是否需要其他相关资源或支持？ | What do you think of the training offered by Rici staff members? How helpful was the training for you to teach the class? Does your school offer any additional resources?  您如何看待日慈员工所提供的课程培训？ 您觉得这些培训对教师实际授课有多大帮助？ 您觉得是否需要其他相关资源？您学校是否有任何其他相关资源或支持？ |

**VI. Implementation (2 questions) 实施（2题）**

| **CFIR Construct** | **Teachers Who Implemented the SEL program**  **教授课程的老师** | **Administrative staff or leaders in the school**  **学校负责的行政人员** |
| --- | --- | --- |
|  | Now, let’s discuss some of your experiences with teaching the SEL classes  现在，我们来聊聊您实施该课程的经验 | Now, let’s discuss the process of how your school implemented the SEL program  现在，我们来聊聊您学校实施该课程的经验 |
| Tailoring Strategies  调整策略 | What kinds of strategies did you take to facilitate the implementation?  您在授课中使用了哪些策略来帮助自己更好开展课程？ | What kinds of strategies did you take to facilitate the implementation within school?  您在学校中使用了哪些策略来帮助授课教师开展课程？ |
| Reflecting & Evaluating  反思 & 评估 | Is there an evaluation system in place in your school for this program?  您学校是否有针对这个课程的评估？  How would you know if the program is effective？  你是如何知道这个课程的有效性呢？ | Is there an evaluation system in place in your school for this program?  您学校是否有针对这个课程的评估？  How would you know if the program is effective？  你是如何知道这个课程的有效性呢？ |

**VII. Characteristics of Individuals (3 questions)**

| **CFIR Construct** | **Teachers Who Implemented the SEL program**  **教授课程的老师** | **Administrative staff or leaders in the school**  **学校负责的行政人员** |
| --- | --- | --- |
|  | Now, I would like to discuss your perceptions of individuals involved within your school and how they can influence the provision of the SEL program  现在，我想了解一下您对学校内部人员的看法，以及他们如何影响了该课程实施 | Now, I would like to discuss your perceptions of the individuals involved within your organization and how they can influence the provision of the SEL program  现在，我想了解一下您对学校内部人员的看法，以及他们如何影响了该课程实施 |
| Leaders  领导 | Who are the key influential individuals to get on board with this implementation? To what extent will they influence teachers’ use of the intervention? The success of the implementation?  您觉得哪些学校内部人员会对该课程实施产生重要影响？ 他们在多大程度上影响了您教授课程及成效？ | Who are the key influential individuals to get on board with this implementation? To what extent will they influence teachers’ use of the intervention? The success of the implementation?  您觉得哪些学校内部人员会对该课程实施产生重要影响？ 他们在多大程度上影响了课程实施及成效？ |
| Capability  能力 | How confident do you feel in your abilities to teach the SEL curriculum?  您对自己教授该课程有多大信心？  Any changes before training versus after training?  参与课程培训是否对您教授该课程的信心产生任何影响？ | How confident do you feel in your abilities to implement the SEL intervention?  您对自己教授该课程有多大信心？ |
| Motivation  动力 | How motivated are you in teaching the SEL classes? Can you explain why?  您参与教授该课程的积极性如何？为什么？ | How motivated are you in implementing the SEL intervention in your school? Can you explain why?  您在学校实施该课程的积极性如何？为什么？ |
| Innovation Recipients | What is about students’ level of buy in?  学生对于课程的接纳和反馈如何？ | What is about students’ level of buy in?  学生对于课程的接纳和反馈如何？ |

**VIII. Conclusion (3 questions) 结语（3题）**

| **Teachers Who Implemented the SEL program**  **教授课程的老师** | **Administrative staff or leaders in the school**  **学校负责的行政人员** |
| --- | --- |
| What are the biggest lessons you’ve learned thus far from teaching the SEL sessions?  到目前为止，您在教授该课程中所收获的最大经验或教训是什么？ | What are the biggest lessons you’ve learned thus far from implementing the SEL program in your school?  到目前为止，您在教授该课程中所收获的最大经验或教训是什么？ |
| What would you describe as your biggest achievements related to teaching the SEL sessions? What would you describe as some of the greatest challenges?  在教授该课程中，让您感受到最有成就感的是什么？ 您认为教授该课程所面临的最大挑战是什么？  what challenges they have encountered that was not expected during planning (the surprise) ？  有哪些挑战是您之前所未曾预料到的？ | What would you describe as your biggest achievements related to implementing the SEL program? What would you describe as some of the greatest challenges?  在教授该课程中，让您感受到最有成就感的是什么？ 您认为教授该课程所面临的最大挑战是什么？  what challenges they have encountered that was not expected during planning (the surprise) ？  有哪些挑战是您之前所未曾预料到的？ |
| Did we miss anything? Do you have anything else you would like to share with me that you did not get to say earlier?  您还有什么需要补充的吗？ | Did we miss anything? Do you have anything else you would like to share with me that you did not get to say earlier?  您还有什么需要补充的吗？ |
